# Making sense of complex data: a mapping process for analyzing findings of a realist review on guideline implementability

**DOI:** 10.1186/1471-2288-13-112

**Published:** 2013-09-12

**Authors:** Monika Kastner, Julie Makarski, Leigh Hayden, Lisa Durocher, Ananda Chatterjee, Melissa Brouwers, Onil Bhattacharyya

**Affiliations:** 1 St. Michael’s Hospital Li Ka Shing Knowledge Institute, 209 Victoria Street, Toronto M5B 1W8, ON, Canada; 2 Department of Oncology, Juravinski Hospital and Cancer Centre, McMaster University, 711 Concession Street, Hamilton L8V 1C3, ON, Canada

## Abstract

**Background:**

Realist reviews offer a rigorous method to analyze heterogeneous data emerging from multiple disciplines as a means to develop new concepts, understand the relationships between them, and identify the evidentiary base underpinning them. However, emerging synthesis methods such as the Realist Review are not well operationalized and may be difficult for the novice researcher to grasp. The objective of this paper is to describe the development of an analytic process to organize and synthesize data from a realist review.

**Methods:**

Clinical practice guidelines have had an inconsistent and modest impact on clinical practice, which may in part be due to limitations in their design. This study illustrates the development of a transparent method for organizing and analyzing a complex data set informed by a Realist Review on guideline implementability to better understand the characteristics of guidelines that affect their uptake in practice (e.g., clarity, format). The data organization method consisted of 4 levels of refinement: 1) extraction and 2) organization of data; 3) creation of a conceptual map of guideline implementability; and 4) the development of a codebook of definitions.

**Results:**

This new method is comprised of four steps: data extraction, data organization, development of a conceptual map, and operationalization vis-a-vis a codebook. Applying this method, we extracted 1736 guideline attributes from 278 articles into a consensus-based set of categories, and collapsed them into 5 core conceptual domains for our guideline implementability map: *Language, Format, Rigor of development, Feasibility, Decision-making*.

**Conclusions:**

This study advances analysis methods by offering a systematic approach to analyzing complex data sets where the goals are to condense, organize and identify relationships.

## Background

Complex interventions, such as those used to improve quality of health care, are informed by principles from health services research, management, psychology and engineering, in addition to medicine. Despite this, they often lack a clear theoretical basis, making it hard to summarize this disparate literature in a way that can inform intervention design or interpretation of results [1]. A realist review is a knowledge synthesis methodology pioneered by Ray Pawson [2], which seeks to better understand what works for whom, in what circumstances and why [2]. Realist reviews are an emerging method with few published examples [3-5], and are particularly relevant for complex and under-conceptualized topics with a heterogeneous evidence base where traditional systematic reviews would often conclude that there is no evidence to inform next steps [6]. The recently published publication standards for Realist Reviews (i.e., RAMESES criteria [7] will likely facilitate improved reporting of this method, as existing techniques to organize and synthesize such information are not well operationalized [8], and require further development to be optimized and to help novice researchers manage large datasets.

To advance the science of analyzing complex and disparate data, this paper describes the development of a process for organizing and analyzing complex evidence in the context of a Realist Review in the area of guideline implementability. We selected guideline implementability to illustrate our data analysis process because guidelines are considered an important knowledge translation tool yet its potential to facilitate the implementation of evidence into clinical practice has largely been unrealized [9-11]. Poor guideline uptake may be due to external factors such as the complex and competing demands on providers’ time, organizational constraints, and lack of knowledge; as well as characteristics of the guidelines themselves (i.e., intrinsic factors). Approaches to improving uptake of guidelines have largely focused on complex knowledge translation interventions consisting of extrinsic strategies that target providers or practice environments. However, these strategies have yielded modest improvement with variable costs [12,13]. Intrinsic strategies (e.g., addressing the clarity, specificity and clinical applicability of recommendations) are promising because they are inexpensive, easy to implement and may be broadly applicable. Additionally, strategies that are being developed do not include disciplines outside of medicine (e.g., management and psychology), so they are not being optimized to advance knowledge in this area. We therefore conducted a realist review to better understand the concept of guideline implementability from a broad perspective of the literature, and to identify how guidelines could be optimized to increase their impact. More specifically, our goal was to identify guideline attributes that affect guideline uptake in clinical practice. The complete protocol for this review is described elsewhere [14], and the final results of this review will be published in a separate paper. Briefly, the realist review considered evidence from four disciplines (medicine, psychology, management, and human factors engineering) to determine what works for whom, in what circumstances and why in relation to guideline implementation [14]. The search strategy included expert-identified, purposive and bibliographic searching. The analytic approach drew on multiple analysis methods (i.e., Realist synthesis and other qualitative synthesis methods). Although the realist review synthesis methods were helpful for interrogating our underlying theory (i.e., why guidelines are not being implemented) [1], Realist Review methods are relatively new, and it’s guidance on the *process* for organizing and relating findings (i.e., the RAMESES criteria [7]) may be a challenge to reproduce by people who are new to the field.

To address this issue, we describe the development of a process for organizing and analyzing complex evidence derived from findings of our realist review on guideline implementability as a means to advance the science of knowledge synthesis.

## Methods and results

**Figure 1:**
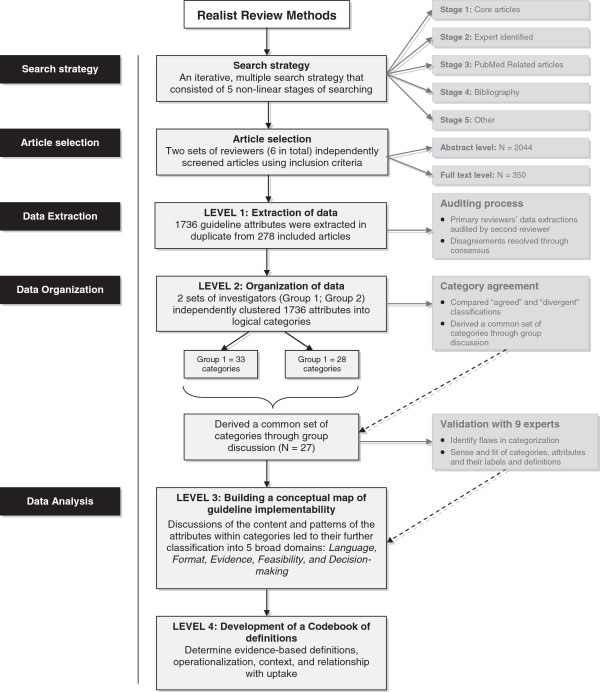
Flow of data analysis process.

Figure 1 shows the flow of the process that was used to make sense of the realist review data consisting of 4 levels of refinement: 1) extraction and 2) organization of data; 3) creation of a conceptual map of guideline implementability; and 4) the operationalization of the map and its components vis a vis the development of a codebook of definitions that will inform the design of a framework. In this section we provide a description of the method used at each step and the results that emerged when the step was applied to our data set.

### Level 1 – Extraction of data

Two groups of investigators extracted 1736 intrinsic guideline attributes (i.e., characteristics) from 278 included articles on study discipline (i.e., medicine, psychology, management, human factors engineering), attribute name and definition (as documented by authors), attribute operationalization (i.e., an explanation of how the attribute functions within the context of the discipline or study), attribute relationship with uptake, and any potential tradeoffs. To ensure reliability, consistency and accuracy of the data extraction, we used an auditing process whereby secondary reviewers checked data extractions of primary reviewers. Disagreements were resolved through consensus-based group discussions involving all investigators.

### Level 2 – Organization of data

The 1736 identified attributes were sorted with the same name or root (e.g., valid/validity) in an Excel database. Two groups of investigators (6 in total, 3 per group) then took the same list of sorted attributes and independently clustered them into logical categories. This involved a process of building up groups of similar or like attributes (including their synonyms and antonyms) that conceptually “fit” within a larger theme, and creating a label and description for each category. Table 1 describes the operationalization of this process. Categorizations between the two groups were compared for agreement aimed at identifying a common set of categories and their included attributes. This involved documenting “agreed” and “divergent” classifications, and making consensus-based decisions through group discussion. This highly systematic approach allowed for efficient filtering and consolidation of a large and complex dataset.

**Table 1:** Operationalization of the categorization process using the “LANGUAGE” domain as an example

**Goal**	**Steps**	**Example**
**Organize, group, and appropriately label similar or “like” attributes**	1. Group attributes that are antonyms	• Complex/Simple
	2. Group attributes that are synonyms	• Unclear/Confusing
	3. Group attributes with the same root	• Specific/Specificity
		• Validity/Valid
	4. Sort database by attribute	
**Categorize attributes into logical clusters**	5. Are there commonalities among attributes?	The following attributes can be grouped into a category called “Clarity”
		• Unambiguous
		• Precise
	6. Is there a central theme or focus among groups of attributes?	• Specific
**Go through each cluster to determine sense and fit of attributes**	7. Do the attributes belong within the same cluster?	The following categories can be collapsed:
	8. Can they be collapsed?	• “Complexity” with “Information overload”
	9. Use attribute definitions to make these decisions	
		• “Actionability” (e.g., using active voice) with “Wording”
**Develop a definition for clusters**	10. Based on their included attributes and definitions, define and label the cluster	The LANGUAGE domain can be defined as: *The clarity, precision, and specificity of the context and message of the guideline*

### Level 3 – Building a conceptual map of guideline implementability

Using a consensus approach among the two groups of investigators via discussions of the attribute definitions and their similarities and relationships, the final set of 27 categories (Table 2) were further grouped into 5 broad dimensions associated with the uptake or use of guidelines: *Language, Format, Rigor of Development, Feasibility, Decision-making*. Based on the evidence around these domains, we developed broad and common sense definitions for each as well as their included categories, which informed a conceptual map of guideline implementability. The development of this map was guided by a web-based visualization tool, MindMeister (http://www.mindmeister.com), which was used iteratively by all investigators to determine the structure of the framework (i.e., moving back-and-forth from the map to definitions and source material), and to facilitate the decision-making process for grouping and identifying patterns in the data. Such visualization techniques have been shown to facilitate comprehension, identify the inferences about the qualities of parts and the relations among them, and be useful for revealing the hierarchy of groupings and important relationships [15].

**Table 2:** Final list of attribute categories across 5 domains of guideline implementability

**Category (N = 27)**	**Major attributes**	**Domain (N = 5)**
**Clarity**	Ambiguity, Specificity, Vagueness	Language
**Cognitive fluency**	Congruity, Fluency, Schema	
**Complexity**	Complexity, Options, Difficult to understand	
**Wording**	Concision, Embedded propositions	
**Framing**	Relative advantage, Gain-loss frame	Format
**Graphical**	Algorithm, Graphs, Tables	
**Inclusion of specific elements in recommendation**	Elements *(e.g., include harms-benefits, patient information, Boolean operators)*	
**Mode of delivery**	Accessibility, Computability	
**Presentation/Layout/Design**	Visual imagery, Presentation	
**Structure/Organization**	Arrangement,	
**Benefits-harms**	Balance of benefits/harms, Dual viewpoint	Rigor of development
**Credibility**	Credible, Authoritative	
**Reliability/Reproducibility**	Reliable, Reproducible, Explicitness	
**Rigor of development**	Evidence-based, Evidence-linked	
**Strength and quality of recommendations**	Quality of evidence, Strength of evidence, Evidence grading	
**Validity**	Validity, Up-to-date	
**Acceptability**	Acceptability, Fit with decision-making, Perceived usefulness, Visibility	Feasibility
**Actionability**	Actionable, Executable, Operationalizable	
**Adaptability**	Adaptability, Context, Tailoring	
**Feasibility**	Feasibility, Compatibility, Costs, Resources	
**Implementation considerations**	Implementability factors affecting feasibility, Trialability	
**Usability**	Ease of use, Usefulness	
**Clinical significance**	Clinical relevance, Applicability	Decision-making
**Considered judgment**	Appropriateness, Value judgments	
**Flexibility**	Flexibility, Clinical freedom	
**Patient preferences**	Patient involvement/communication/values	
**Values**	Beliefs, Compatibility, Values/Norms	

To validate and to identify potential flaws in categorization and to obtain agreement on the sensibility and fit of attributes within and across the categories, a group of 9 stakeholders with knowledge translation and guideline development expertise were surveyed. These experts were asked to review the content of the 5 domains and its sub-domains, and to rename, rearrange and condense attributes as they saw fit. The survey comprised Likert-type and open-ended questions about the operational definition of the domains, and the fit of categories and their attributes within them *(see Additional file**1**)*. Through consensus-based discussions amongst our team, findings of this survey were used to make modifications to the organization and structure of our data (e.g., collapsing and renaming some attributes, categories and domains).

### Level 4 – Development of a codebook

The two groups collectively developed a codebook of definitions to better understand each of the 5 domains of implementability, the relationships between guideline attributes and their uptake, and potential tradeoffs. The process involved documenting definitions for modifiable attributes (i.e., those that have the potential to be changed by guideline developers) and their operationalization (i.e., how the attribute can be used and examples of how it functions), the context and setting in which these occur, for whom, any relationship with uptake, and attribute tradeoffs if they existed *(see the Additional file**2 **for an example Codebook)*. The codebook was developed one domain at a time using a modified duplicate reviewing process that involved a set of primary reviewers extracting and documenting the information, and a second group of reviewers “auditing” (i.e., checking) primary reviews in small-group discussions; a third group of reviewers resolved disagreements. The main objectives of the auditing process were to verify the completion of documentation, to ensure the appropriate understanding of concepts, and to determine the best fit of attributes and information within and between categories and domains.

## Discussion

Complex interventions are often atheoretical and loosely draw on a broad literature that includes different disciplines and is difficult to summarize systematically. Qualitative synthesis methods are poorly operationalized and do not describe how to organize and analyze large heterogeneous datasets. We used a systematic process of analysis to build a conceptual map of guideline implementability through the classification of 1736 attributes into a consensus-based set of categories, which were then collapsed into 5 core conceptual domains of guideline implementability: *Language, Format, Rigor of development, Feasibility, Decision-making*. These findings will be used to answer our Realist review question: *What is it about guidelines that facilitate or impede their uptake, for whom and in what circumstances this happens, and how and why this happens.*

We reviewed a range of review methods to answer our research. The details explaining the rationale for selecting a Realist Review is published in our protocol [14]. Briefly, we assessed a range of review methods (i.e., Realist Review, Meta-narrative synthesis, and Meta-ethnography) to determine which of these was the most appropriate, but we found that none were a “perfect fit” to sufficiently cover all our questions. We selected the Realist Review method because the approach provides the most systematic guidance on how to conduct a complete review (i.e., a process for a search strategy, article selection, and data analysis), it allows the inclusion of diverse evidence (i.e., quantitative and qualitative), and provides an explanatory investigation of underlying theories and mechanisms of the study under investigation. In our case, ‘causation’ was determined by considering the interaction between contexts (i.e., the circumstances and settings of guideline use), mechanisms (i.e., the processes operating within guidelines that explain why they are used in some circumstances but not in others) and outcomes (whether guidelines are used or not). We theorized that unpacking these C-M-O relationships would facilitate our understanding of guideline implementability. However, one difficulty with the Realist Review method is that it lacks a comprehensive process to compare disciplinary perspectives on a given issue. We then considered Meta-narrative synthesis, which can be helpful for analysing data across different fields or disciplines [16]. Meta-ethnography was another method that we considered, which involves translating key concepts from one study to another to reveal new insights [17], but its application to large data sets and its focus on qualitative studies presents challenges when the data set is large and comprised of mixed study designs. This lack of a “perfect fit” highlights the need to consider all factors associated with the research question when deciding which method is the most appropriate to answer them. These included determining the breadth of evidence needed (quantitative or qualitative or both) and balancing this need with the feasibility or resources available to perform the review, anticipating the end-users of findings, and to what extent the method provides strategies for rigor and transparency. In fact, these are similar considerations we may use for selecting the most appropriate methods for primary studies. There has been a resurgence of interest in developing new knowledge synthesis methods to address the limitations of some of the traditional synthesis strategies such as the systematic review. Like realist review, the advantage of these methods is that they can help organize information from underconceptualized fields like knowledge translation and quality improvement to create a more cumulative knowledge base. However, methodological strategies that are more accessible are required if they are to be widely used and optimized. To this end, a scoping review by Tricco *et al.* is currently underway to determine which knowledge synthesis methods are available, and to develop a systematic process to help researchers select the most appropriate method(s) to address their research questions about complex evidence [18].

A limitation of our work is that the approach we used was largely interpretive. However, the quality of synthesis is dependent on reviewers’ explicitness and reflexivity of the methods. In our process to make sense of the complex data that emerged from our Realist Review, we ensured transparency of the methods and included several validity measures to minimize sources of error. This was important given the interpretive nature of our process and the anticipated learning curve involved in data abstraction. The measures included an auditing process whereby primary data extractions was checked by secondary reviewers, and a process to verify this data against a codebook of definitions during Level 4 analysis. Lastly we tested the validity of our data organization and analysis through an expert survey to verify the sense and fit of attributes and categories within the framework.

In our realist review, we considered each attribute and integrated like-attributes into common themes and domains. Further, we considered evidence of impact or effectiveness on our relevant outcome. For example, evidence indicates that a guideline recommendation is more actionable if it clearly specifies when, who should do precisely what action; if a recommendation does not specify these steps or uses passive verbs, its actionability will be diminished. Such conceptualization of the evidence can then be useful to support or refute various theories or their elements in the literature about guideline implementability. These strategies enabled us to embrace the whole of the data, with few preconceived expectations, to identify and carefully define elements that are relevant to guideline uptake. The approach described in this paper is an example of how new analytic methods can emerge and respond to the challenges related to finding the best fit between methods and research questions. Based on our experience, Table 3 highlights suggested steps to help determine the purpose and scope of poorly understood concepts under investigation such as guideline implementability. This may be particularly useful to help organize, synthesize, validate, and represent complex data resulting from qualitative reviews in a relevant and meaningful way.

**Table 3:** Suggested approach to organize, synthesize, validate and make sense of complex findings

**Step**	**Points to consider**	**Example**	**Advantages**	**Challenges**	**How to overcome challenges**
**1. Selection of analysis method**	• Which method is the most appropriate to answer research questions?	• We searched the literature for various synthesis methods of complex evidence	• Potentially more valid if the method matches the question	• There was no single synthesis method that best fit our questions	• Need to adopt a flexible approach to match appropriate methods to answer research questions
					• Consider selecting a primary analysis method supplemented by other or modified methods to address all questions
**2. Organization and analysis of data**	• How will the data be organized?	• We sorted and organized our data *(1736 guideline attributes)* in an Excel database	• Sorting of concepts and themes on multiple levels *(e.g., across attributes, categories, disciplines)*	• Difficult to keep track of changes from multiple reviewers	• We used a modified duplicate review process that involved a group of second reviewers “auditing” the analysis of primary reviewers
					• Ensure that document tracking is transparent and efficient *(e.g., track and document changes and include detailed notes from all reviewers)*
				• Duplicate review is time consuming and resource intensive	
		• Analysis process was done in duplicate	• Duplicate analysis minimizes bias		
	• Will also depend on selected analysis method				
**3. Validity measures**	• How are you going to verify findings and minimize bias?	• Sought expert consensus on findings using survey methodology	• Survey methodology is quick and efficient	• Survey methodology has inherent biases	• Depending on resources, other consensus methods may increase validity such as the Delphi method
					• Transparency *(i.e., document what was planned, what was done and why)*
**4. Representation of data**	• How will the results and data be used?	• We developed a conceptual map of guideline implementability for guideline developers and end-users	• The conceptual map contributes to the understanding of guideline implementability	• There may be other factors not captured in the map that may influence guideline implementability	• The conceptual framework needs to be refined according to the codebook of definitions
					• The conceptual framework needs to be rigorously evaluated to determine the feasibility of its use by guideline developers, and its potential to influence guideline uptake by family physicians
			• The process advances the knowledge about analysis methods for complex evidence		
	• Who are the target knowledge end users?				
**5. Dissemination of data**	• To what extent should the data be disseminated?	• The map will inform a guideline implementability framework for guideline developers, users and policy makers	• The framework will inform end-users about attributes that facilitate guideline uptake; and may also inform policy around guideline development	• There may be other factors influencing guideline implementability	• Prior to dissemination, the framework will need to undergo rigorous evaluation *(including quantitative and qualitative studies)* to test its potential to influence guideline uptake by family physicians who are the primary end-users of clinical practice guidelines
	• Will the work inform practice, system, policy?				

Our work has the potential for wide influence. The proposed method will appeal to more investigators because the process has now been operationalized, is fairly straightforward to apply, it can be applied to a wide range of topics and the return on effort is significant. Expanding this knowledge base will become particularly important as these rapidly expanding fields most often require more sophisticated techniques to analyze data, which is informed by complex interventions that cut across multiple disciplines and from the input of multiple stakeholders.

## Conclusions

This study represents a novel contribution to advancing complex data analysis methods by offering a systematic approach to analyzing any large and disparate data sets where the goals are to condense, organize and identify relationships.

## Competing interests

None of the authors have any financial or non-financial competing interests to declare.

## Authors’ contributions

All authors contributed in the design of the study. MK, LH, AC, JM, LD executed the study and MK, LH, JM, AC, LD, OB, MB conducted the analysis and interpreted the results. MK drafted the manuscript, and all authors read and approved the final manuscript.

## Pre-publication history

The pre-publication history for this paper can be accessed here:

http://www.biomedcentral.com/1471-2288/13/112/prepub

## Supplementary Material

Additional file 1 Expert feedback review form on the Guideline Implementability Framework. Click here for file

Additional file 2 Example of a Codebook of definitions. Click here for file
