# Supplementary material for: Making sense of complex data: a mapping process for analyzing findings of a realist review on guideline implementability
Source: BMC Med Res Methodol. 2013 Sep 12;13:112. doi: 10.1186/1471-2288-13-112 (PMC3848005; doi:10.1186/1471-2288-13-112)
Supplement: Additional file 1 — Expert feedback review form on the Guideline Implementability Framework. [file 1471-2288-13-112-S1.doc]

**Additional file 1**

Expert feedback review form on the Guideline Implementability Framework

| **Questions** | | **Feedback** | |
| --- | --- | --- | --- |
| **Big picture review** *(please answer the following questions with the “complete” framework in mind)* | | | |
|  | When you open this up, the framework splits into 1) Creation of content; and 2) Communication of content – the logic behind this was how we conceptualized guideline development occurring in the real world, and where in this process the domains might fit | |  |
|  | Does this split make sense? | |  |
|  | Are you happy with the way our 5 domains are spread across them? | |  |
| **Domain level review** *(please answer the following questions with the 5 individual domains in mind: Rigor of Development, Feasibility, Considered Judgment, Format, and Language). For each domain, look at the sub-categories and how they are organized)* | | | |
| **RIGOR OF DEVELOPMENT** | Does the domain make sense? | |  |
|  | Do the labels make sense and appropriately convey the domains/subcategories? | |  |
|  | Some of the domains have numbered subcategories because we think there is logic to their order (FORMAT and RIGOR) – For these, does this order make sense? | |  |
| **FEASIBILITY** | Does the domain make sense? | |  |
|  | Do the labels make sense and appropriately convey the domains/subcategories? | |  |
|  | Some of the domains have numbered subcategories because we think there is logic to their order (FORMAT and RIGOR) – For these, does this order make sense? | |  |
| **CONSIDERED JUDGMENT** | Does the domain make sense? | |  |
|  | Do the labels make sense and appropriately convey the domains/subcategories? | |  |
|  | Some of the domains have numbered subcategories because we think there is logic to their order (FORMAT and RIGOR) – For these, does this order make sense? | |  |
| **FORMAT** | Does the domain make sense? | |  |
|  | Do the labels make sense and appropriately convey the domains/subcategories? | |  |
|  | Some of the domains have numbered subcategories because we think there is logic to their order (FORMAT and RIGOR) – For these, does this order make sense? | |  |
| **LANGUAGE** | Does the domain make sense? | |  |
|  | Do the labels make sense and appropriately convey the domains/subcategories? | |  |
|  | Some of the domains have numbered subcategories because we think there is logic to their order (FORMAT and RIGOR) – For these, does this order make sense? | |  |
| **ADDITIONAL COMMENTS** | | | |
