# Supplementary figures and images for: Making sense of complex data: a mapping process for analyzing findings of a realist review on guideline implementability
Source: BMC Med Res Methodol. 2013 Sep 12;13:112. doi: 10.1186/1471-2288-13-112 (PMC3848005; doi:10.1186/1471-2288-13-112)

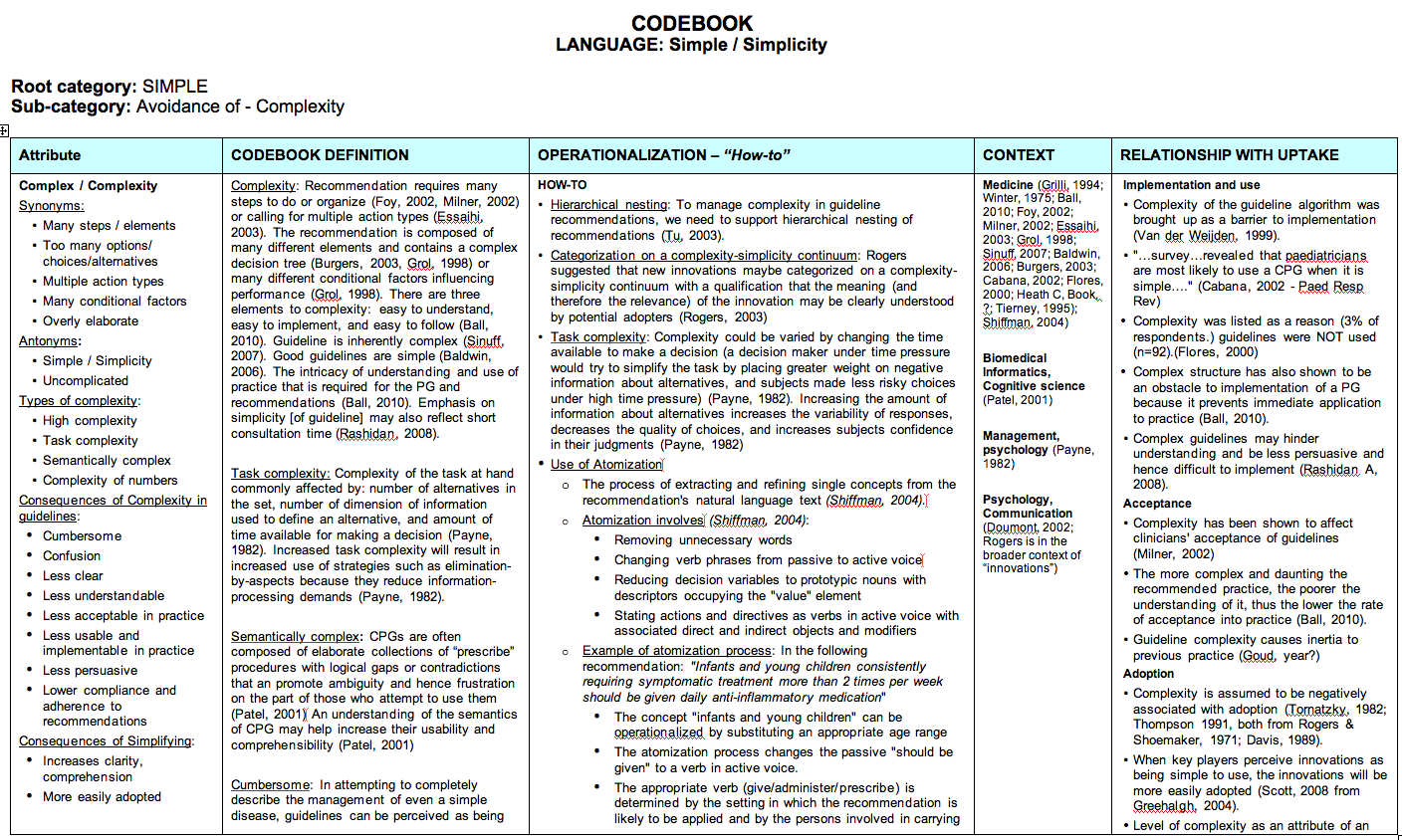

Supplement: Additional file 2 — Example of a Codebook of definitions. [file 1471-2288-13-112-S2.tiff]
